# A multi-source remote sensing and machine learning framework for maize mapping and yield estimation in fragmented Loess gully regions

**DOI:** 10.3389/fpls.2026.1892566

**Published:** 2026-07-01

**Authors:** Lei Sun, Hao Li, Shangkun Li, Shuanghui Zhao, Yanqun Zhang, Yan Mo, Baozhong Zhang, Youjie Wu

**Affiliations:** 1 College of Water Resources & Civil Engineering, Hunan Agricultural University, Changsha, China; 2 State Key Laboratory of Water Cycle and Water Security, China Institute of Water Resources and Hydropower Research, Beijing, China; 3 Department of Irrigation and Drainage, China Institute of Water Resources and Hydropower Research, Beijing, China; 4 School of Soil and Water Conservation, Beijing Forestry University, Beijing, China

**Keywords:** crop mapping, multi-source feature fusion, SHAP, smallholder farming, yield estimation

## Abstract

Accuracy crop distribution mapping and reliable yield estimation are essential for overcoming fragmentation and decentralization in smallholder farming systems of the Loess Plateau gully region. Multi-source remote sensing data, ancillary datasets, and machine learning techniques were integrated to map maize distribution and estimate yield. First, Sentinel-2 temporal spectral features, vegetation indices, and topographic variables were integrated to identify the optimal maize mapping model by a comparing machine learning algorithms: Random Forest (RF), Extra Trees (ET), Gradient Boosting Decision Tree (GBDT), and Histogram-Based Gradient Boosting Decision Tree (HGBDT). Subsequently, Sentinel-2 optical data and ERA5-Land meteorological data were dynamically resampled and spatiotemporally fused. A maize yield estimation model was then developed by integrating these fused predictors with *in-situ* measured maize yield samples. Finally, SHapley Additive exPlanations (SHAP) analysis was applied to quantify the feature contribution to both crop mapping and yield estimation, improving model transparency and interpretability. The results indicate that RF model achieved superior performance for maize identification in heterogeneous agricultural landscapes, with an overall Accuracy of 0.825, Precision of 0.849, Recall of 0.933, and F1-Score of 0.889. In the multi-source fusion-based yield estimation task, the HGBDT model yielded the highest predictive accuracy, with an R² of 0.6, RMSE of 1.07 t/ha, relative RMSE (rRMSE) of 11.49%, and MAE of 0.86 t/ha. Here, a methodological advancement is presented toward accurate and interpretable crop mapping and yield estimation in the ecologically complex and topographically fragmented Loess Plateau.

## 1 Introduction

Global population growth and increasing frequent climate events create uncertainly for crop production (Hu et al., 2023) Accurate crop yield prediction is therefore critical for ensuring food security, optimizing agricultural policy, and guiding food production systems (Miao et al., 2024). Maize is a major staple and feed crop worldwide, its yield stability directly affects regional grain self-sufficiency. However, in the typical hilly-gully region of the Loess Plateau, fragmented smallholder plots, spectral mixing, and highly decentralized farm management practices collectively generate complex agro-ecological conditions. These factors hinder accuracy maize mapping and large-scale yield estimation (Xiong et al., 2026; Xu et al., 2023).

Satellite remote sensing (RS), with its high temporal frequency and broad spatial coverage, has become a widely used approach for monitoring dynamic crop growth (Blickensdörfer et al., 2022; Hu et al., 2019; Ozdarici-Ok et al., 2015). Time series vegetation indices (VIs) derived from high resolution imagery, such as Sentinel-2, can effectively characterize crop phenological development (Peng et al., 2023). For instance, Khan et al. (2025) developed a multi-stream deep neural network using the Normalized Difference Vegetation Index (NDVI) and Enhanced Vegetation Index (EVI) time series for county-scale yield prediction; Guo and Ren (2023) demonstrated that red-edge spectral features improve the separability between maize and forest–grassland mixtures under spectrally complex backgrounds; Meng et al. (2025) proposed a multidimensional feature framework integrating spectral, structural, and textural information, substantially enhancing the robustness of crop biomass estimation. Furthermore, Qiu et al. (2025) fused Sentinel-1 and Sentinel-2 time-series data to construct a dual-driven crop identification framework, achieving accurate mapping of multiple crop types in smallholder farming systems. Multi-source data fusion has been shown to reduce estimation biases induced by plot fragmentation. Zhou et al. (2024) reported that multi-source data fusion reduces prediction errors by up to 40%, however, their study was conducted in flat, homogeneous terrain. Lu et al. (2024) developed a deep learning framework for yield prediction and confirmed that integrating complementary data sources improves accuracy by 15% relative to single-source approaches, yet their analysis focused on large-scale, mechanized farming systems. Therefore, in ecologically complex and topographically fragmented areas such as the gullied regions of the Loess Plateau, how to effectively integrate multi-source features remains an issue that needs to be addressed.

The accuracy of crop spatial extent extraction depends on minimizing misclassification between crops and natural vegetation. However, most machine learning frameworks for regional-scale mapping still use the default classification probability threshold of 0.5 to generate final outputs (Long et al., 2024). Xie and Niculescu (2022) found, that fixed low-confidence thresholds exacerbate noise induced by spectral overlap among land cover types. Similarly, Romano et al. (2025) reported substantial commission errors at patch-edge pixels when applying the default threshold in land cover mapping of fragmented landscapes in Italy, leading to systematic deviations between mapped area statistics and ground-truth field survey data. Despite these empirical findings, there remains a lack of a systematic method to calibrate the optimal classification confidence threshold in fragmented smallholder farming systems to ensure the reliability of county-level area estimates.

Current ensemble learning models primarily focus on enhancing predictive accuracy, and their inherent black-box nature impedes mechanistic understanding of the biophysical processes governing yield formation (Padarian et al., 2019; Pant et al., 2025). In the ecologically distinct Loess Plateau, conventional prediction models struggle to explicitly elucidate how diverse feature variables, spanning spectral, phenological, and meteorological domains, collectively drive the spatial differentiation of maize yield (Muhammad et al., 2026). Mohan et al. (2025) integrated SHAP (Shapley Additive Explanations) and LIME (Local Interpretable Model-agnostic Explanations) into a yield prediction framework, successfully quantifying the marginal contributions of key environmental drivers to crop yield, thereby establishing a mechanistic foundation for interpreting spatial yield variability. Paudel et al. (2023) systematically investigated the interpretability of deep learning models in crop yield forecasting, employing SHAP to disentangle the relative importance of remote sensing and meteorological features; their findings confirmed that eXplainable AI (XAI) methods can effectively demystify black-box model behavior. Likewise, several independent studies (Malashin et al., 2024; Snehalatha and Patil, 2026) have further corroborated the robustness and interpretive utility of SHAP in crop yield prediction. Thus, SHAP enables precise quantification of multi-dimensional feature contributions, while revealing both local feature effects and global mechanisms through which environmental factors shape yield heterogeneity, thereby forging a scientifically grounded bridge between data-driven modeling and dryland agronomy (Chen et al., 2026; Lundberg and Lee, 2017; Smith et al., 2026).

Therefore, we developed a remote sensing–based framework for maize mapping and yield estimation by integrating multi-source datasets with machine learning in Pengyang County, Ningxia Province, China, a representative case of the Loess Plateau’s hilly and gully region. The specific objectives were: (1) to achieve high-accuracy maize mapping and reliable county-level area statistics by fusing Sentinel-2 time-series spectral and phenological features and optimizing machine learning classifiers with adaptive confidence thresholds to address fragmented land cover and spectral mixing in the Loess hilly region; (2) to construct a county-scale yield estimation framework by synergistically integrating optical remote sensing, meteorological, and topographic data and adopting a task-oriented strategy for machine learning model selection; and (3) to elucidate the agronomic mechanisms driving maize distribution and yield variability in complex environments by incorporating SHAP interpretability to quantify the marginal contributions of climate, terrain, and phenology to maize identification and yield formation.

## 2 Study area and data

### 2.1 Study area

**Figure 1:**
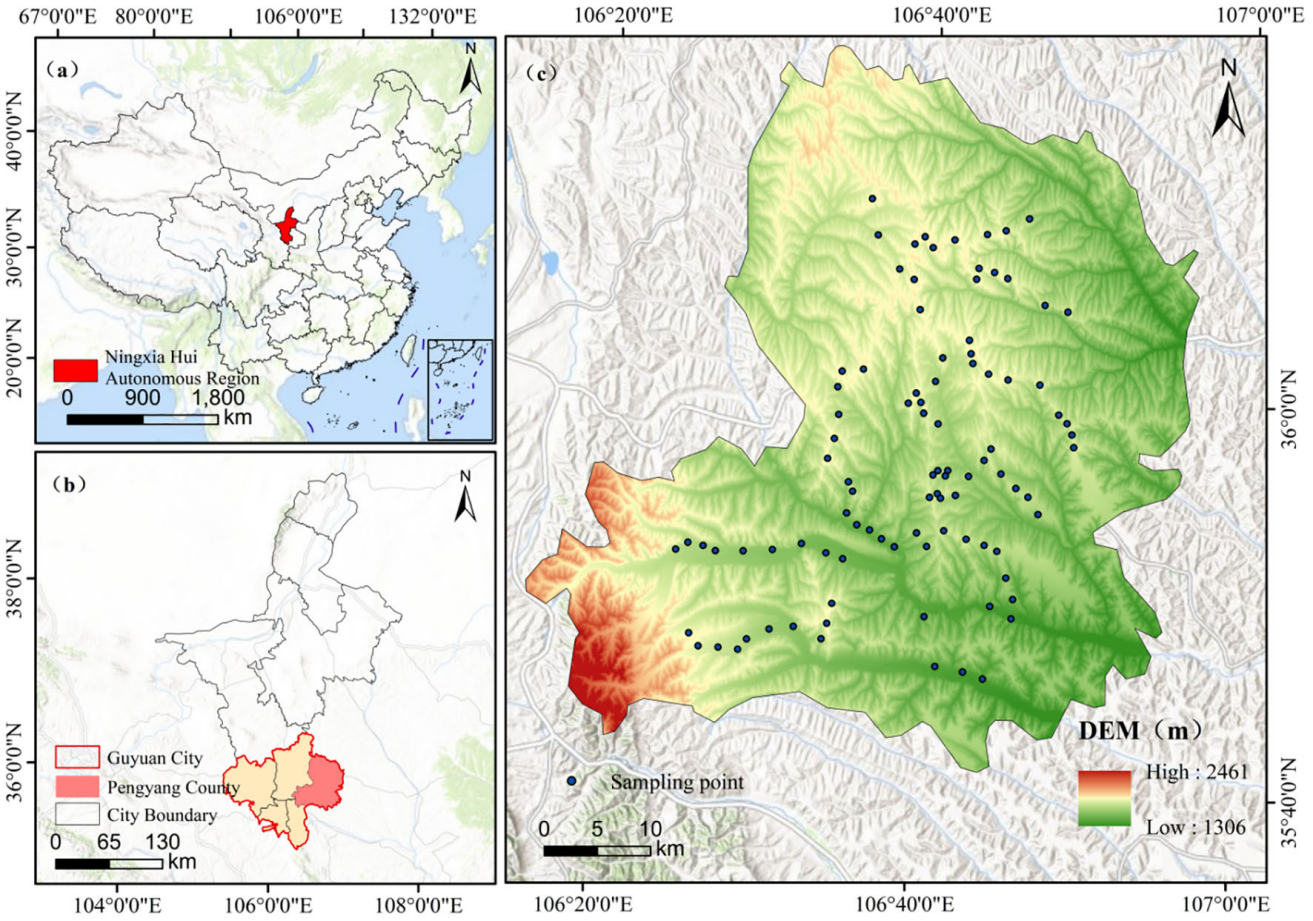
Overview of the study area. **(A)** Geographic location of the Ningxia Province within China; **(B)** location of Pengyang County within the Ningxia Province; **(C)** topographic representation of the study area derived from a digital elevation model (DEM), overlaid with yield sampling points.

The study area is Pengyang County in the southeastern Ningxia Province China (**Figure 1**). It lies between 106°32′–106°58′ E and 35°41′–36°17′ N, covering an area of 2.533 × 10^3^ km². The region exhibits a temperate semi-arid continental monsoon climate, with mean annual precipitation ranging from 500 to 700 mm. However, precipitation is highly seasonal and spatially variable, leading to pronounced intra-annual drought patterns.

According to the 2025 Statistical Bulletin of National Economic and Social Development of Pengyang County, the total sown area for grain crops was 4.98 × 10^4^ ha, of which maize accounted for 3.02 × 10^4^ ha, representing 61% of the total grain crop area. Maize is typically sown from late April to early May, reaches heading and grain-filling stages from mid-July to mid-August, and is harvested in October.

### 2.2 Data and preprocessing

#### 2.2.1 Sentinel-2 imagery

This study employed high-resolution optical remote sensing data from Sentinel-2 satellite imagery, which offers both high temporal resolution (a 5-day revisit cycle) and high spatial resolution (up to 10 m). Leveraging the Google Earth Engine (GEE) cloud platform (Gorelick et al., 2017), we acquired all available Sentinel-2 Level-2A Surface Reflectance (SR) products covering the study area during the critical maize growth period in 2025 (April–October). These Level-2A products are radiometrically calibrated and atmospherically corrected, ensuring robust spectral fidelity for subsequent analysis. We first filtered out images with cloud cover exceeding 10% using the built-in CLOUDY_PIXEL_PERCENTAGE metadata property and applied a rigorous cloud masking procedure. Monthly median composites were then generated to derive time-series reflectance values for the visible bands (B2, B3, B4), the near-infrared band (B8), and the shortwave infrared bands (B11, B12) (Papadopoulou et al., 2023). This composite dataset demonstrated exceptional stability and discriminative capability for agricultural land cover classification. In order to ensure spatial consistency across all spectral bands, the 20 m-resolution shortwave infrared bands were uniformly resampled to 10 m using bilinear interpolation (**Table 1**).

**Table 1:** Spatial and temporal resolutions of the datasets used in this study.

Dataset	Native spatial resolution	Used spatial resolution	Temporal resolution
Sentinel-2 (L2A)	10 m (B2, B3, B4, B8); 20 m (B11, B12)	10 m	5 d
ERA5-Land	1000 m	10 m	1 h
SRTM	30 m	10 m	Static

#### 2.2.2 Meteorological data

Meteorological data were acquired from the ERA5-Land hourly reanalysis dataset, developed and disseminated by the European Centre for Medium-Range Weather Forecasts (ECMWF) (Hersbach et al., 2020). Specifically, we extracted the following variables over the maize growing season (April–October): monthly mean air temperature, monthly cumulative precipitation, and monthly mean surface solar radiation. All meteorological raster datasets were then resampled to a uniform spatial resolution of 10 m to match that of the optical imagery, using dynamic boundary matching during the localization processing stage (**Table 1**).

#### 2.2.3 Topographic data

Topographic auxiliary data were acquired from the NASA Shuttle Radar Topography Mission (SRTM) Version 3.0 Digital Elevation Model (DEM). In the hilly region of the Loess Plateau with pronounced topographic relief, a high-resolution, geometrically accurate DEM is essential to mitigate topographic shadow effects and enable robust identification and delineation of actual agricultural cropland in complex mountainous terrain. The SRTM dataset was accessed via the GEE platform; elevation and slope layers were then derived for the study area and resampled to a 10 m spatial resolution to ensure geometric consistency with the optical base map (**Table 1**).

#### 2.2.4 Ground sample data

A total of 130 ground reference points were selected across the county, including 100 maize and 30 non-maize samples. These samples served two primary purposes: (i) Crop mapping samples, three field surveys were conducted during the periods of 26–31 July, 26–31 August, and 26–30 September 2025. During each survey, maize and non-maize land covers were visually identified and geolocated using GPS, with latitude and longitude coordinates recorded for each point. Sentinel-2 image acquisition dates corresponding to the three *in-situ* ground measurements are listed in **Supplementary Table 1**. (ii) Yield estimation samples, 96 maize sample plots were selected to ensure plot dimensions exceeded 10 × 10 m. Within each plot, five 2 × 2 m sub-quadrats were established: one at the plot center and one at each of the four diagonal corners. At physiological maturity, maize plants within each sub-quadrat were harvested manually, threshed, and air-dried to a moisture content of approximately 14%. Grain weight was measured and converted to standard yield (t/ha) using standardized conversion protocols (**Table 2**).

**Table 2:** Statistical characteristics of measured maize yield data across 96 sampling sites.

Maximum yield (t/ha)	Minimum yield (t/ha)	Mean yield (t/ha)	Coefficient of variation (CV)
14.55	4.99	9.89	0.2

## 3 Methodology

### 3.1 Feature construction

Spectral features of remote sensing imagery are fundamental to image interpretation. In addition to the six original reflectance bands, we computed three biophysically meaningful spectral indices, the Normalized Difference Vegetation Index (NDVI), which characterizes overall canopy development; the Green Chlorophyll Vegetation Index (GCVI), which is particularly sensitive to early-stage chlorophyll content; and the Land Surface Water Index (LSWI), which serves as an indicator of canopy water stress (Wei et al., 2023).

In response to the spectral confusion commonly induced by single-date imagery, we comprehensively examined the dynamic growth trajectory of maize across its entire growing season, from April to October, and developed four advanced phenological metrics based on time-series NDVI: peak greenness during the growing season (NDVI_Max); minimum background reflectance (NDVI_Min); mean greenness over the season (NDVI_Mean); greenness amplitude (NDVI_Amp). Among these, NDVI_Amp effectively captures the pronounced biophysical progression of maize, spanning from bare-soil sowing in spring, through canopy closure in summer, ending in senescence in autumn (Han H. et al., 2025). As such, it serves as a robust discriminant feature for distinguishing cultivated cropland from natural vegetation.

Using the 18 features listed in **Table 3** as the candidate feature pool, we employed SelectFromModel with a RandomForestClassifier (classification) and RandomForestRegressor (regression) (n_estimators = 100, random_state = 42) to rank and select the top 12 features via mean decrease in impurity (MDI). The identical selected feature subset was applied uniformly across all four models in each task to prevent model-specific selection bias and ensure comparability.

**Table 3:** Selected feature variables and their calculation formulas.

Feature category	Feature variable	Description or calculation formula	Reference
Spectral features	B2	Sentinel-2 Level-2A surface reflectance	
	B3	Sentinel-2 Level-2A surface reflectance	
	B4	Sentinel-2 Level-2A surface reflectance	
	B8	Sentinel-2 Level-2A surface reflectance	
	B11	Sentinel-2 Level-2A surface reflectance	
	B12	Sentinel-2 Level-2A surface reflectance	
Spectral index features	NDVI	$\frac{\text{B}8-\text{B}4}{\text{B}8+\text{B}4}$	(Tucker, 1979)
	GCVI	$\frac{\text{B}8}{\text{B}3}-1$	(Gitelson et al., 2003)
	LSWI	$\frac{\text{B}8-\text{B}11}{\text{B}8+\text{B}11}$	(Xiao et al., 2004)
Temporal phenological features	NDVI Max	Max (NDVIt), where t⊆ [4,10] (months)	
	NDVI Min	Min (NDVIt), where t⊆[4,10] (months)	
	NDVI Mean	$\frac{1}{n}\sum_{t=4}^{10}NDVIt$	
	NDVI Amp	NDVI Max−NDVI Min	
Topographic features	Elevation	Surface elevation obtained from SRTM	
	Slope	Surface slope angle calculated from SRTM elevation data	
Meteorological features	Temperature	Monthly mean temperature for April–October obtained from ERA5-Land	
	Precipitation	Monthly total precipitation for April–October obtained from ERA5-Land	
	Radiation	Monthly mean net solar radiation for April–October obtained from ERA5-Land	

### 3.2 Crop mapping and yield estimation model selection

#### 3.2.1 Random forest

Random Forest (RF) is an ensemble machine learning method rooted in the Bagging (Bootstrap Aggregating) framework (Breiman, 2001). RF aggregates predictions from multiple trees to improve accuracy and robustness. Each tree is trained on a bootstrapped sample and a random subset of features, introducing randomness at two levels. This dual-source randomness effectively mitigates model variance, reduces susceptibility to overfitting, and improves generalization performance. For the yield estimation regression task addressed in this study, the final predicted value y is computed as the arithmetic mean of the individual predictions generated by all constituent decision trees (Equation 1):

(1) $$\overset{⌢}{\text{y}}=\frac{1}{\text{T}}\sum_{t=1}^{T}f_{t}(x)$$

Here, f_t_(x) denotes the prediction output of the t decision tree, and T represents the total number of trees in the ensemble. The RF model demonstrates strong robustness to high-dimensional data and outliers, enabling it to effectively capture complex nonlinear relationships between multi-source remote sensing features and crop-related targets.

#### 3.2.2 Extra trees

Extra Trees (ET) is an extension and variant of RF, and also a decision tree–based ensemble learning algorithm (Geurts et al., 2006). However, it introduces a higher degree of randomization during node splitting. Unlike RF, which selects a random subset of training samples and then searches for the optimal split point over a random subset of features, the ET constructs each tree using the entire training set and randomly samples both features and split thresholds at each node, without optimizing for impurity reduction. This enhanced randomization further mitigates overfitting and reduces the model’s sensitivity to individual training instances. As with RF, the final prediction is obtained by aggregating the outputs of all constituent trees (Equation 2):

(2) $$\overset{⌢}{y}=\frac{1}{T}\sum_{t=1}^{T}f_{t}(x)$$

Here, f_t_(x) denotes the prediction output of the t tree in the ET ensemble, and T represents the total number of trees. Compared with conventional ensemble methods, the ET algorithm generally achieves greater variance reduction and higher computational efficiency, thereby offering distinctive robustness to noise, particularly when analyzing complex geographical data characterized by strong background noise in the Loess Plateau.

#### 3.2.3 Gradient boosting decision trees

Gradient Boosting Decision Trees (GBDT) constitute an iterative ensemble method for decision trees (Friedman, 2001), grounded in the boosting framework, which enhances predictive accuracy by sequentially combining multiple weak learners into a single strong learner. In contrast to RF, which construct trees independently and in parallel, GBDT builds trees sequentially, each subsequent tree is trained to approximate the negative gradients of the loss function with respect to the predictions of the current ensemble, effectively modeling the discrepancies between the outputs of the preceding model and the true target values. By iteratively fitting new trees along the steepest descent direction of the loss function, the algorithm progressively refines its approximation of the underlying mapping between inputs and outputs. The final prediction is expressed as a weighted sum of all constituent trees (Equation 3):

(3) $$F_{T}(x)=\sum_{t=1}^{T}\gamma_{t}h_{t}(x)$$

Here, h_t_(x) denotes the m base regression tree, T represents the total number of boosting iterations, and denotes the weight assigned to the t tree. By sequentially correcting the residual errors of preceding models, the GBDT achieve exceptionally high feature utilization and strong fitting capacity, making it particularly well-suited for modeling heterogeneous spatial features characterized by large-scale variations and multicollinearity.

#### 3.2.4 Histogram-based gradient boosting decision trees

Histogram-Based Gradient Boosting Decision Tree (HGBDT) is a highly efficient, optimized variant of conventional GBDT, specifically designed to accelerate training on large-scale, high-dimensional datasets (Ke et al., 2017). Its core innovation lies in discretizing continuous floating-point feature values into a fixed number of discrete integer bins and constructing decision trees using precomputed feature histograms, rather than performing exact sample-wise sorting at each node. This histogram-based approach significantly reduces both the computational complexity and memory overhead associated with identifying optimal split points. Consequently, information gain computation during node splitting can be performed efficiently using aggregated histogram statistics (Equation 4):

(4) $$Gain=\frac{1}{2}(\frac{(\sum g_{L}{)}^{2}}{\sum h_{L}+\lambda }+\frac{(\sum g_{R}{)}^{2}}{\sum h_{R}+\lambda }-\frac{(\sum g{)}^{2}}{\sum h+\lambda })$$

Here, g and h denote the sums of the first-order and second-order gradients, respectively, aggregated within each histogram bin; L and R represent the left and right child nodes resulting from a split; and λ is the regularization penalty parameter. By integrating histogram-based gradient approximation into the boosting framework, HGBDT not only preserves the high predictive accuracy characteristic of gradient boosting algorithms but also significantly mitigates the computational bottleneck associated with training large-scale models, particularly in regional-scale remote sensing mapping and crop yield estimation tasks involving massive datasets.

#### 3.2.5 Model parameter settings for crop mapping

For the RF classifier, the number of decision trees (n_estimators) was set to 150 to ensure stable ensemble predictions. The maximum tree depth was strictly constrained to 4 (max_depth = 4) to prevent spatial overfitting in the spectrally heterogeneous and fragmented landscape of the Loess Plateau gully region. The minimum samples required at leaf nodes and for internal node splitting were set to 5 (min_samples_leaf = 5) and 10 (min_samples_split = 10), respectively, to avoid fitting isolated noisy pixels while maintaining sufficient model capacity. The ET model was configured with identical values of n_estimators (150), max_depth (4), min_samples_leaf (5), and min_samples_split (10) to ensure a fair comparison under consistent model complexity constraints.For the GBDT, the number of boosting stages (n_estimators) was set to 150, with max_depth limited to 4 and min_samples_leaf set to 5 to control model complexity in the heterogeneous terrain. The learning rate was set to 0.1 (learning_rate = 0.1) to prevent rapid overfitting during sequential residual correction. The HGBDT was configured with max_iter = 150 (functionally equivalent to n_estimators in other models), max_depth = 4, and learning_rate = 0.1, maintaining comparable complexity to GBDT while leveraging histogram-based acceleration for efficient large-scale raster prediction.

#### 3.2.6 Model parameter settings for yield estimation

For the RF regressor, the number of decision trees was set to 200 (n_estimators = 200), the maximum tree depth to 8 (max_depth = 8), the minimum number of samples at leaf nodes to 3 (min_samples_leaf = 3). The ET regressor was configured with identical parameters to the RF model (n_estimators = 200, max_depth = 8, min_samples_leaf = 3, random_state = 42, n_jobs = -1) to ensure a fair comparison under consistent model complexity constraints.For the GBDT regressor, the number of boosting stages was set to 150 (n_estimators = 150), the maximum tree depth to 4 (max_depth = 4), the learning rate to 0.05 (learning_rate = 0.05), and the subsample ratio to 0.7 (subsample = 0.7). The relatively shallow tree depth and conservative learning rate were intentionally set to mitigate overfitting risks in heterogeneous spatial features, while the subsample strategy introduced stochasticity to further reduce model variance. For the HGBDT regressor, the maximum number of iterations was set to 150 (max_iter = 150, functionally equivalent to n_estimators in GBDT), the maximum tree depth to 4 (max_depth = 4), and an L2 regularization penalty was applied (l2_regularization = 0.5) to alleviate overfitting in the high-dimensional multi-source feature space, while leveraging the histogram-based acceleration mechanism to ensure computational efficiency for large-scale raster inversion.

### 3.3 Model development for crop mapping and yield estimation

**Figure 2:**
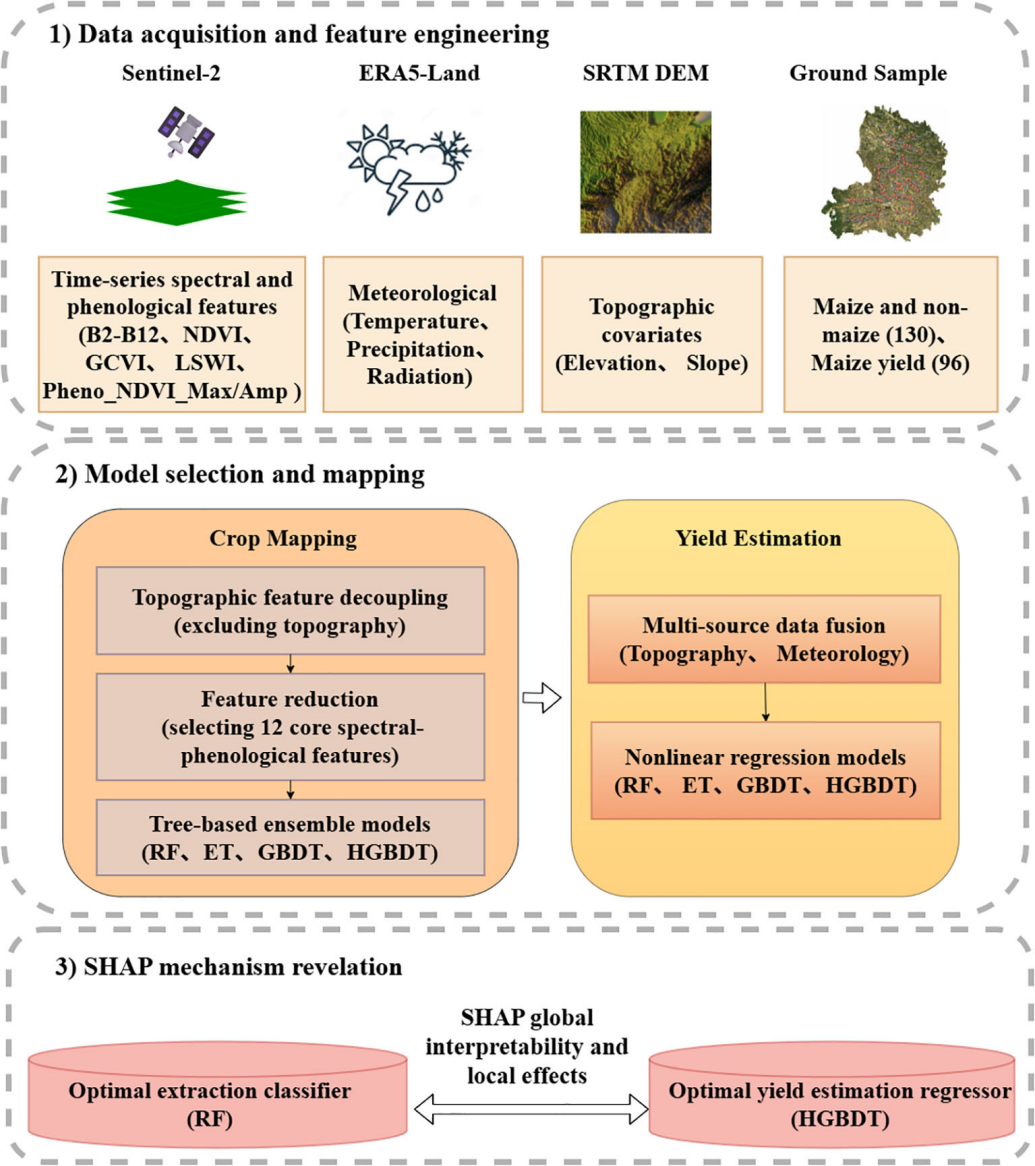
Experimental workflow.

The experimental workflow, built upon the synergistic integration of the GEE cloud platform and a local Python environment, is illustrated in **Figure 2**.

**Figure 3:**
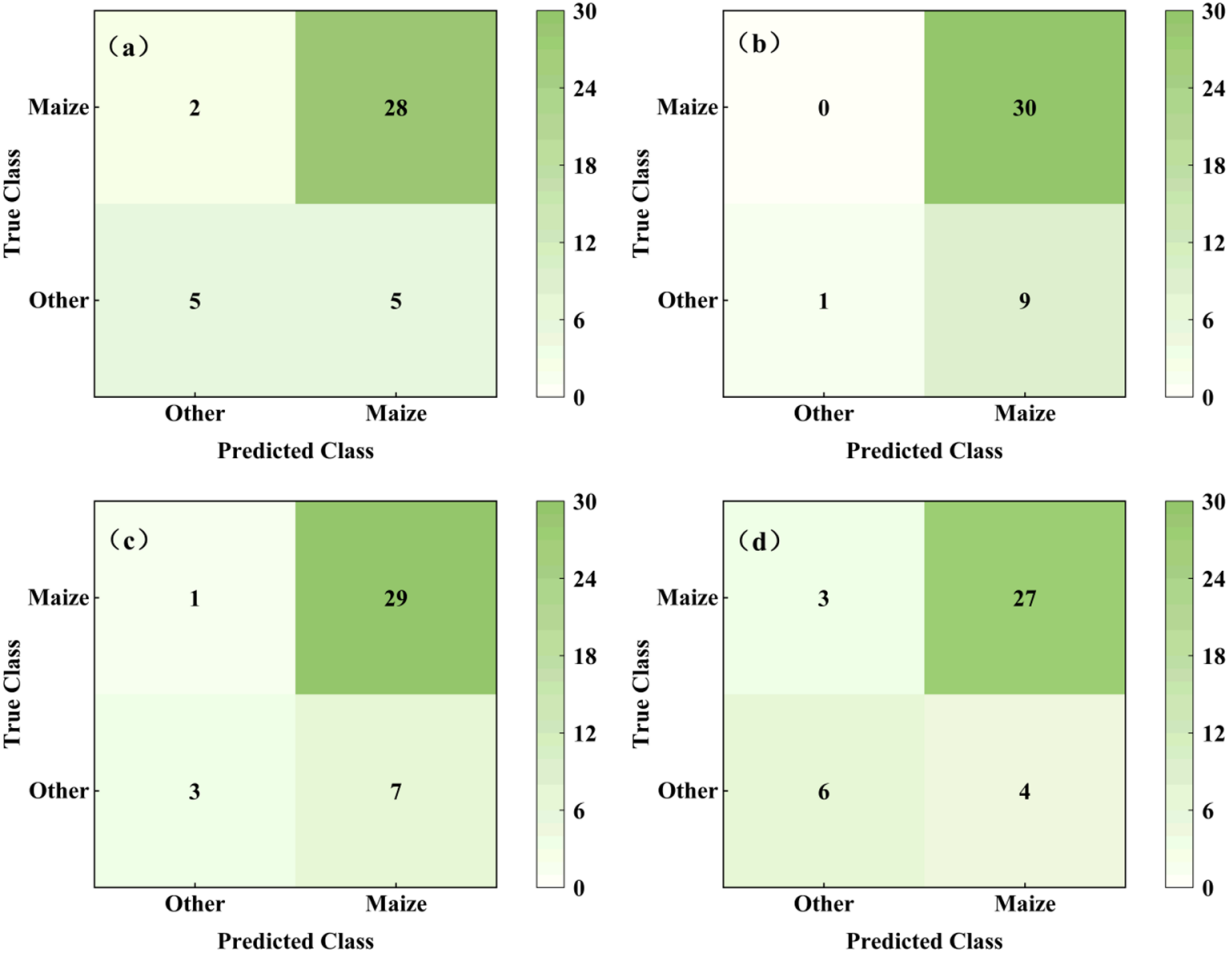
Confusion matrix evaluation of the models. **(A)** Random Forest (RF); **(B)** Extra Trees (ET); **(C)** Gradient Boosting Decision Trees (GBDT); **(D)** Histogram-Based Gradient Boosting Decision Tree (HGBDT).

1. Sentinel-2 Level-2A surface reflectance imagery spanning the entire maize growing season was acquired through the GEE platform. Cloud masking was applied to remove contaminated pixels, followed by monthly median compositing to generate cloud-free, temporally consistent image mosaics. From these composites, spectral indices, including the NDVI, GCVI, and LSWI, were computed. In addition, temporal phenological features were extracted from the multi-temporal index time series. Topographic features were derived from the SRTM digital elevation model. Meteorological data were acquired from the ERA5-Land hourly reanalysis dataset.
2. The maize and non-maize classification samples were randomly split into training and validation sets at a 7:3 ratio. Meanwhile, the 96 *in-situ* maize yield measurements were partitioned into training and validation sets at an 7:3 ratio. After extracting the corresponding multi-source features for each pixel, feature dimensionality reduction was performed using the SelectFromModel method.
3. The classification training set was used to train and tune hyperparameters for four machine learning models: RF, ET, GBDT, and HGBDT. Among these, RF achieved the best classification performance and was therefore selected as the optimal classifier. Preliminary experiments revealed that the default threshold of 0.50 produced severe overestimation of maize area in the fragmented Loess Plateau landscape. With the aim of systematically identifying the optimal threshold while maintaining computational efficiency, we evaluated a gradient of thresholds from 0.50 to 0.85 in steps of 0.05. The results for the full gradient (0.50, 0.55, 0.60, 0.65, 0.70, 0.75, 0.80, 0.85) confirmed that area statistics progressively converged toward ground-truth values as the threshold increased. For clarity of presentation, **Supplementary Figure 1** (0.50, 0.55,0.60, 0.85) and **Figure 3** displays the four most informative thresholds (0.65, 0.70, 0.75, 0.80). At each threshold level, the relative error between the model-predicted maize area and the official statistics reported in the statistical yearbook was computed. The threshold yielding the minimum relative area error, 0.80, was identified as optimal. Finally, the entire Pengyang County raster image was processed pixel-wise using the optimized RF model to generate the 2025 maize spatial distribution map and to estimate the total planting area.
4. Using the maize-only mask generated in Step (3), optical features (10 m resolution) and meteorological features (1,000 m resolution) were spatially resampled to a common 10 m grid and subsequently fused. These integrated multi-source features were then input into the selected machine learning model to predict absolute yield values for each maize pixel, yielding a final county-scale spatial yield distribution map.

### 3.4 Model accuracy evaluation

For the purpose of comprehensively and objectively assessing the classification accuracy of crop mapping and the reliability of yield estimation, this study established two distinct accuracy evaluation frameworks, one for crop mapping and another for yield estimation.

#### 3.4.1 Classification accuracy metrics

For the purpose of rigorously evaluating the classification accuracy of the extracted crop maps, we employed four standard metrics, Accuracy, Precision, Recall, and F1-Score, derived from the confusion matrix. These metrics collectively provide a comprehensive assessment of model performance in crop-type mapping. The corresponding calculation formulas are as follows (Equations 5–8):

(5) $$Accuracy=\frac{TP+TN}{TP+TN+FP+FN}$$

(6) $$\text{Precision}=\frac{\text{TP}}{\text{TP}+\text{FP}}$$

(7) Recall=TPTP+FN

(8) F1-Score=2×Precision−RecallPrecision+Recall

Here, TP denotes the number of maize samples correctly classified as maize; TN denotes the number of non-maize samples correctly classified as non-maize; FP denotes the number of non-maize samples incorrectly classified as maize; and FN denotes the number of maize samples incorrectly classified as non-maize.

#### 3.4.2 Yield estimation accuracy metrics

For the purpose of rigorously assessing the predictive performance of the yield estimation models, four quantitative evaluation metrics were adopted: the coefficient of determination (R^2^), root mean square error (RMSE), relative root mean square error (rRMSE), and mean absolute error (MAE). Higher R^2^ values and lower values of RMSE, rRMSE, and MAE collectively indicate superior model accuracy. The corresponding mathematical formulations are as follows (Equations 9–12):

(9) $$R^{2}=1-\frac{\sum_{i=1}^{n}{\left(y_{i}-{\hat{y}}_{i}\right)}^{2}}{\sum_{i=1}^{n}{\left(y_{i}-{\bar{y}}_{i}\right)}^{2}}$$

(10) $$RMSE=\sqrt{\frac{1}{n}\sum_{i=1}^{n}{\left(y_{i}-{\hat{y}}_{i}\right)}^{2}}$$

(11) $$rRMSE=\frac{RMSE}{\hat{y}}\times 100\%$$

(12) $$MAE=\frac{1}{n}\sum_{i=1}^{n}\left|y_{i}-{\hat{y}}_{i}\right|$$

Here, n denotes the total number of samples in the measured yield validation set; ${\text{y}}_{\text{i}}$ represents the experimentally measured yield value of the sample; ${\hat{\text{y}}}_{\text{i}}$ denotes the yield value predicted by the model; and $\hat{\text{y}}$ denotes the mean of the measured yield values across all samples.

#### 3.4.3 Landscape metrics for classification threshold assessment

For the purpose of objectively evaluating the spatial pattern quality and areal fidelity of the maize classification under varying confidence thresholds, five quantitative landscape metrics were adopted: the class area (CA), relative error (RE), mean patch area (AREA_MN), percentage of like adjacencies (PLADJ), and aggregation index (AI). The corresponding mathematical formulations are as follows (Equations 13–17):

(13) $$CA=\sum_{i=1}^{n}a_{i}$$

Here $a_{i}$ is the area of patch i, and n is the total number of maize patches.

(14) $$Relative\;Error=\frac{CA_{estimated}-CA_{oficial}}{CA_{official}}\times \;100\%$$

(15) $$AREA\_MN=\frac{\sum_{i=1}^{m}a_{i}}{n}=\frac{CA}{n}$$

(16) $$PLADJ=(\frac{g_{ii}}{\sum_{k=1}^{m}g_{ik}})\times 100\%$$

Here $g_{ii}$ is the number of like adjacencies (maize-to-maize) and $\sum_{k=1}^{m}g_{ik}$ is the total number of adjacencies involving maize pixels.

(17) $$AI=(\frac{g_{ii}}{max(g_{ii})})\times 100\%$$

Here $max(g_{ii})$ is the maximum possible number of like adjacencies for a given class area under a compact spatial configuration.

## 4 Results

### 4.1 Performance comparison of different models for maize crop mapping

We evaluated the performance of RF, ET, GBDT and HGBDT models for crop mapping using four standard classification metrics of Accuracy, Precision, Recall, and F1-Score (**Table 4**). In terms of Accuracy, RF and HGBDT achieved identical scores of 0.825, followed by GBDT (0.800) and ET (0.775). For Precision, HGBDT attained the highest value (0.871), outperforming RF (0.849), GBDT (0.806), and ET (0.769). Recall exhibited a distinct pattern, ET achieved a perfect score of 1, substantially higher than all other models, whereas GBDT, RF, and HGBDT yielded Recall values of 0.967, 0.933, and 0.9, respectively. With respect to the F1-Score, RF achieved the highest value (0.889), closely followed by HGBDT (0.885), GBDT (0.879), and ET (0.87). Collectively, these results suggested that the RF model delivered the most balanced performance, achieving the top F1-Score while maintaining high Recall and a strong Precision.

**Table 4:** Performance comparison of Random Forest (RF), Extra Trees (ET), Gradient Boosting Decision Trees (GBDT), Histogram-Based Gradient Boosting Decision Tree (HGBDT) using Accuracy, Precision, Recall, and F1-Score.

Model	Accuracy	Precision	Recall	F1-Score
RF	0.825	0.849	0.933	0.889
ET	0.775	0.769	1.000	0.870
GBDT	0.800	0.806	0.967	0.879
HGBDT	0.825	0.871	0.900	0.885

**Figure 3** presented the confusion matrices of the four models on the test set. Comparison of diagonal (correct) and off-diagonal (incorrect) entries reveals distinct performance patterns. For maize samples, RF model correctly classified 28 out of 30 maize samples, missing only two maize samples (**Figure 3A**). In contrast, the ET, GBDT, and HGBDT models exhibited a progressive decline in correct maize identifications of 30, 29, and 27 samples, respectively, with a corresponding increase in omission errors (**Figures 3B–D**). For non-maize samples, HGBDT model achieved the lowest commission error, misclassifying only four non-maize samples as maize (**Figure 3D**). RF model followed closely with five such errors (**Figure 3A**), whereas GBDT and ET exhibited higher commission errors, with seven and nine misclassifications, respectively (**Figures 3C, B**).

**Figure 4:**
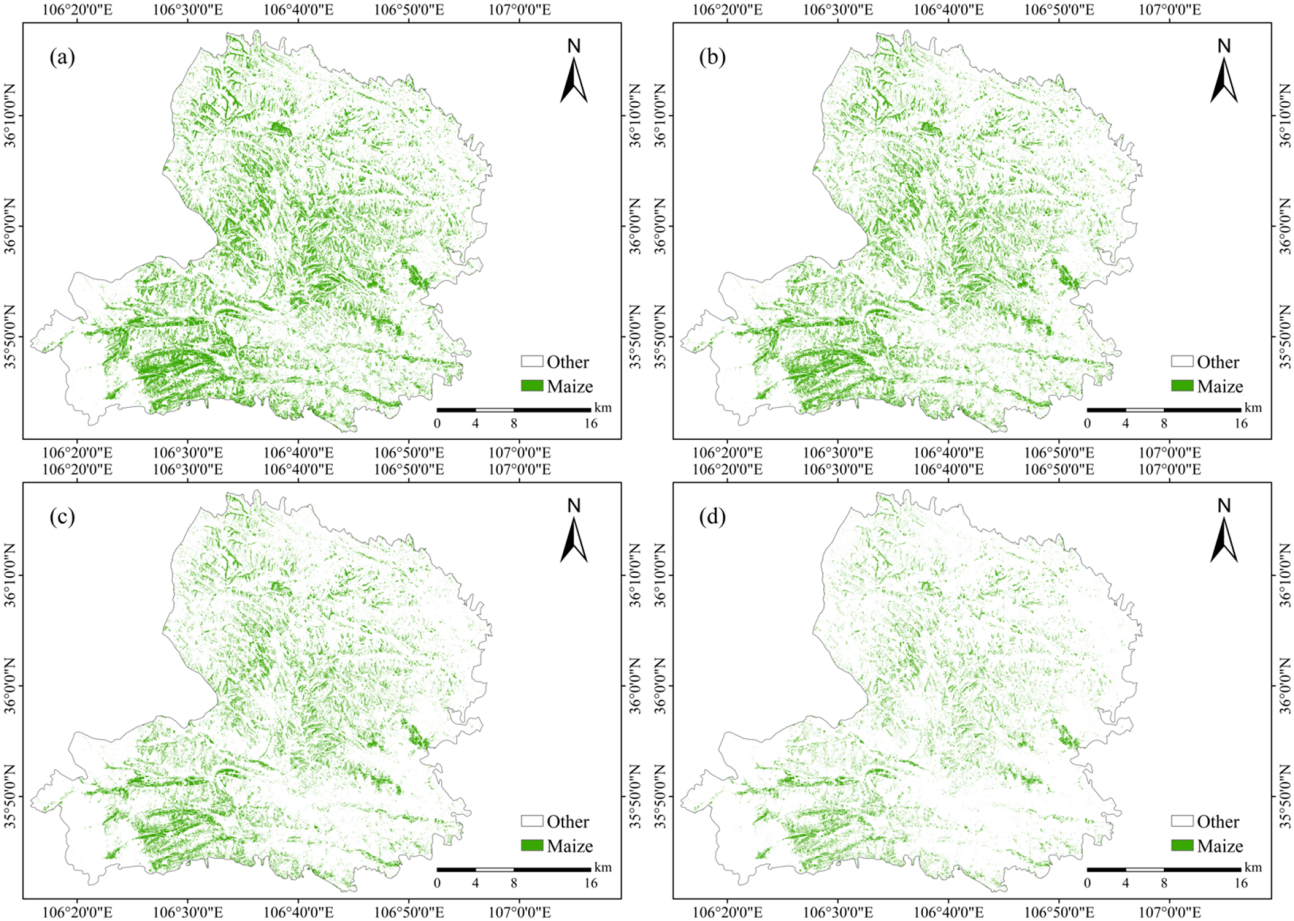
Spatial distribution extraction results obtained under varying threshold values using Random Forest (RF). **(A)** 0.65; **(B)** 0.70; **(C)** 0.75; **(D)** 0.80.

**Figure 4** presented the maize spatial distribution results derived from the optimal classification model of RF under varying classification confidence thresholds (0.65; 0.70; 0.75; 0.80),The spatial distribution maps of maize generated under the remaining classification confidence thresholds (0.50, 0.55, 0.60, and 0.85) are provided in **Supplementary Figure 1**. At the default threshold of 0.65, over half of the fields were classified as maize, a result inconsistent with ground truth (**Figure 4A**). With the goal of improving classification accuracy, we systematically increased the threshold from 0.65 to 0.80 in steps of 0.05 (**Figures 4A–D**). When the confidence threshold was raised to 0.8 (**Figure 3D**), the optimized RF model identified a total of 2.753 × 10^6^ maize pixels across the study area. Based on this high-accuracy spatial classification mask, the total maize planting area in Pengyang County for 2025 was estimated at 2.753 × 10^4^ ha. This estimate deviates by only 8.8% from the official Crop Statistical Yearbook (3.02 × 10^4^ ha), representing the closest agreement among all tested thresholds.

**Table 5** presents the landscape metrics of maize classification under four confidence thresholds. The CA decreases monotonically from 5.787 × 10^4^ ha at 0.65 to 2.754 × 10^4^ ha at 0.80. Correspondingly, the RE decreases from +91.6% to −8.8%, with the 0.80 threshold yielding the closest estimate to the official statistics. The AREA_MN decreases from 0.49 ha to 0.20 ha across the gradient. Similarly, the PLADJ declines from 70.20% to 56.83%, and the AIdeclines from 80.53% to 72.52%.

**Table 5:** Landscape metrics of maize classification under varying confidence thresholds.

Threshold	CA (ha)	RE (%)	AREA_MN (ha)	PLADJ (%)	AI (%)
0.65	5.787 × 10^4^	+91.6	0.49	70.20	80.53
0.70	4.750 × 10^4^	+57.3	0.38	66.23	79.72
0.75	3.589 × 10^4^	+18.9	0.27	61.03	75.84
0.80	2.754 × 10^4^	-8.8	0.20	56.83	72.52

Class Area (CA): The total area of all pixels classified as maize in the study region; Relative Error: The percentage deviation between the estimated maize area and the official statistical yearbook value; Mean Patch Area (AREA_MN): The average area of individual maize patches; Percentage of Like Adjacencies (PLADJ): The proportion of edges shared between adjacent maize pixels relative to the total edges of all maize pixels; Aggregation Index (AI): A measure of the spatial clustering and contiguity of maize patches.

### 4.2 Performance evaluation of multi-source fusion models for maize yield estimation and spatial mapping

**Figure 5:**
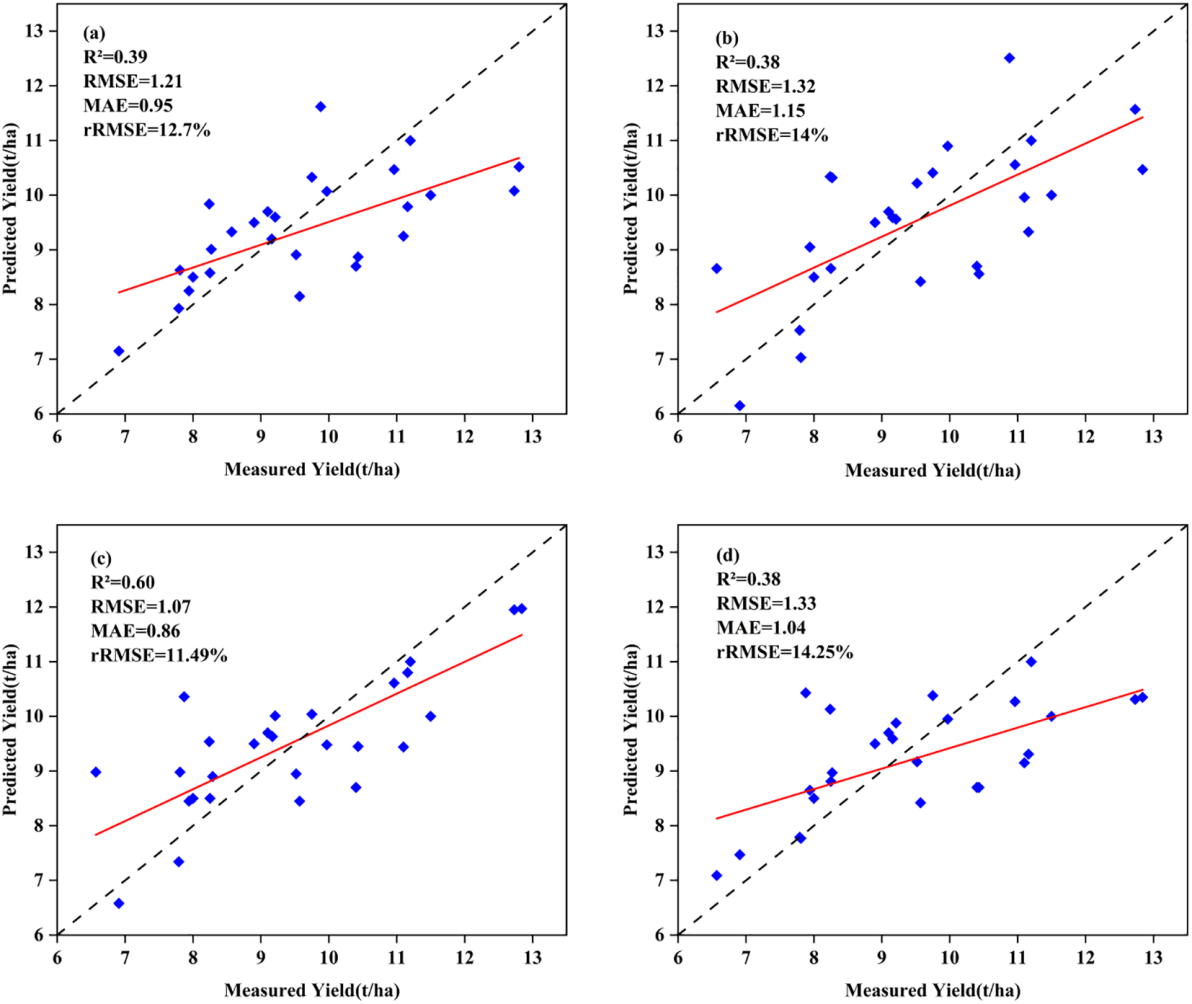
Scatterplots of measured versus model-predicted maize yields. **(A)** Extra Trees (ET); **(B)** Gradient Boosting Decision Trees (GBDT); **(C)** Histogram-Based Gradient Boosting Decision Tree (HGBDT); **(D)** Random Forest (RF).

We then developed a multi-source fusion model for maize yield estimation by integrating spectral, phenological, meteorological, and topographic features based on the derived maize distribution map. We systematically evaluated the yield prediction performance of each model using scatterplots of observed versus predicted yields, and the comparative results are presented in **Figure 5**. The HGBDT model demonstrated the strongest fitting performance, achieving the highest coefficient of determination (R^2^ = 0.6) among all evaluated models, along with the lowest error metrics (RMSE = 1.07 t/ha, rRMSE=11.49%, MAE = 0.86 t/ha) (**Figure 5C**). The scatter points of the HGBDT model clustered more tightly around the 1:1 reference line (black dashed line), and its fitted regression line (red solid line) exhibited the smallest deviation from this reference line. In contrast, the GBDT (**Figure 5B**), ET (**Figure 5A**), and RF (**Figure 5D**) models showed lower fitting accuracy, with R^2^ values of 0.38, 0.39, and 0.38, respectively. Their corresponding error metrics were substantially higher, and their fitted regression lines deviated markedly from the 1:1 reference line, indicating systematic underestimation of high observed values and overestimation of low observed values.

**Figure 6:**
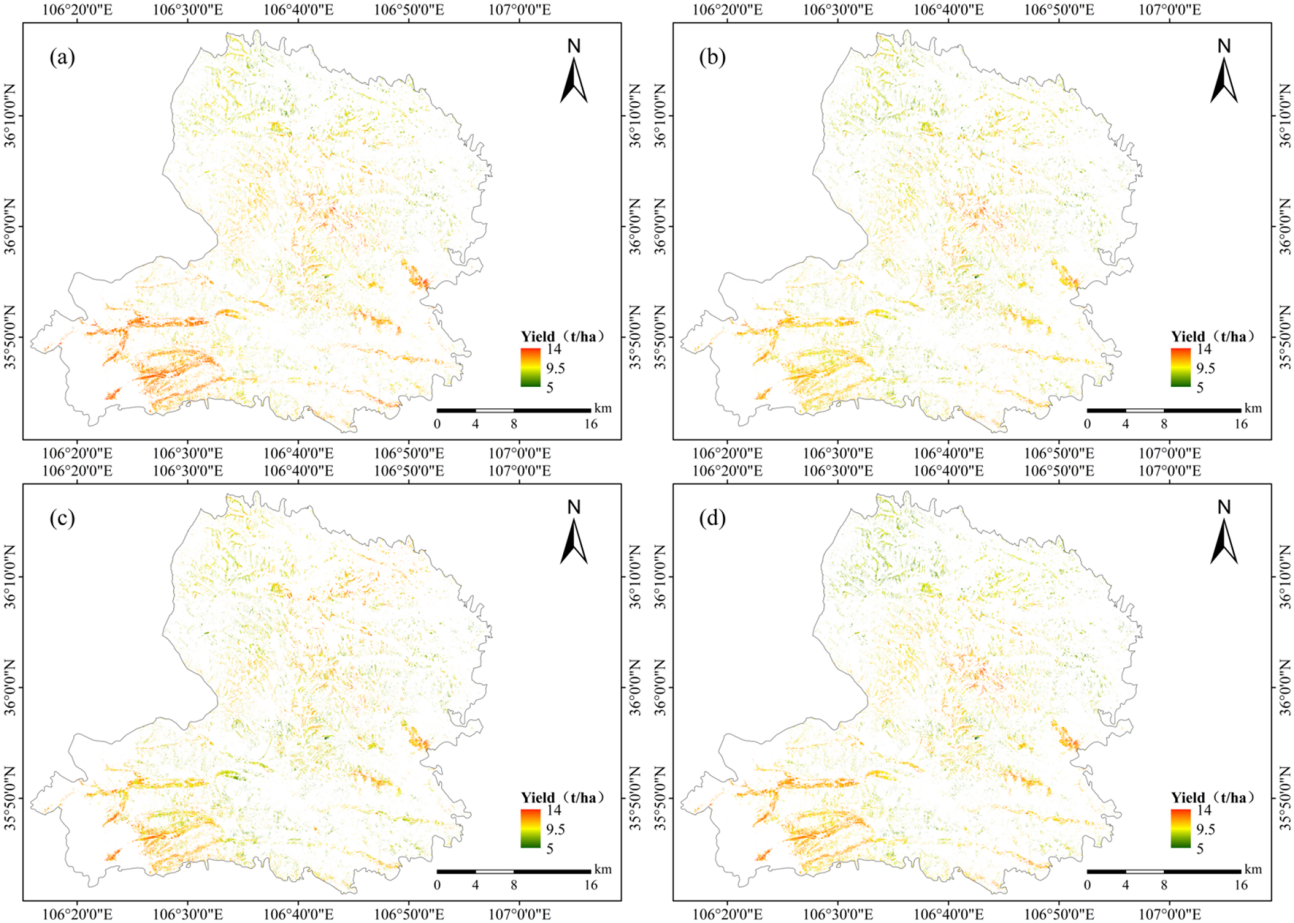
Spatial distribution patterns of predicted maize yield. **(A)** Extra Trees (ET); **(B)** Gradient Boosting Decision Trees (GBDT); **(C)** Histogram-Based Gradient Boosting Decision Tree (HGBDT); **(D)** Random Forest (RF).

**Figure 6** displayed the spatial distribution maps of maize yield predicted by the four yield estimation models. The results indicated substantial spatial variation in estimated maize yield across the study area, ranging from 5 t/ha (green, low yield) to 14 t/ha (red, high yield). Spatially, high-yield areas were predominantly concentrated in the southwestern portion of the study area and exhibited a distinct linear or dendritic spatial extension pattern. In contrast, medium and low-yield areas are primarily located in the northern and northeastern regions, where they occurred in a fragmented, patchy, and interspersed manner without forming large-scale contiguous clusters.

### 4.3 SHAP-based driver analysis for spatial structure extraction and yield estimation of maize

**Figure 7:**
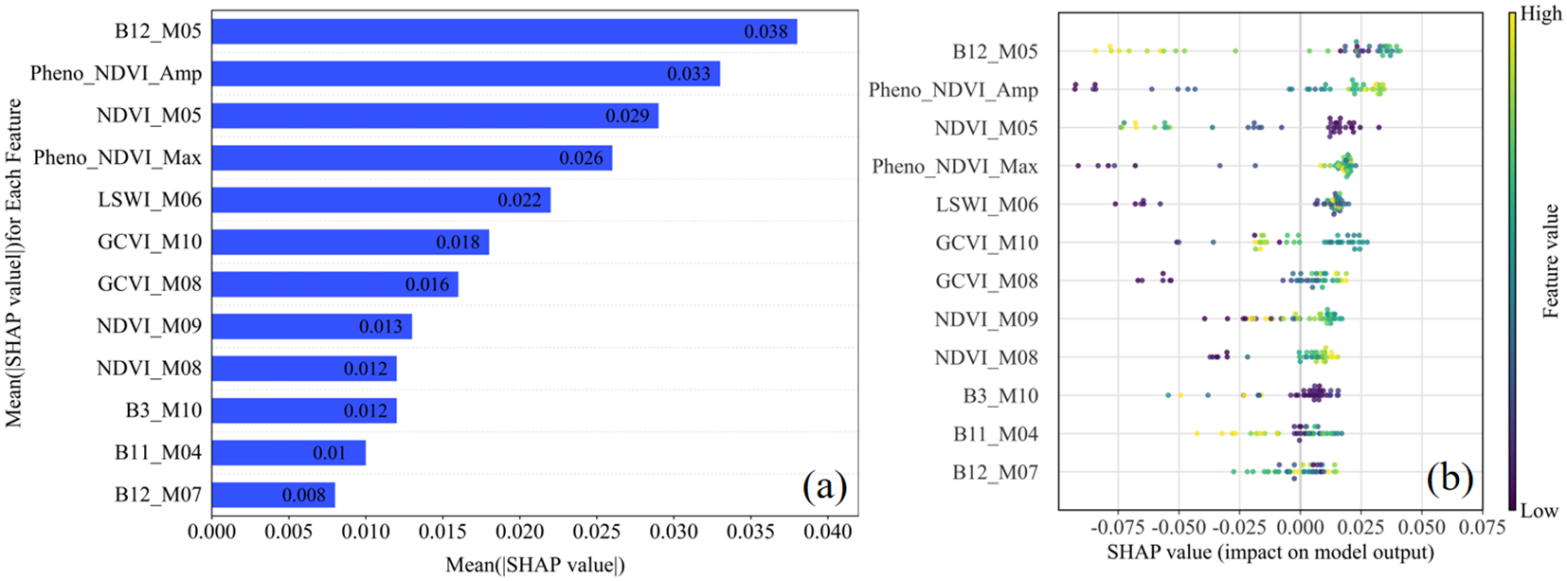
SHAP-based interpretation of feature importance for Random Forest model. **(A)** Global feature importance ranking, computed as the mean absolute SHAP value across all samples. **(B)** SHAP beeswarm plot, showing the distribution of SHAP values for each feature and their influence on model predictions, with points color-coded by feature value (purple: low; yellow: high).

The SHAP value distribution and global feature importance ranking generated by the RF model elucidated the contributions of each feature to maize mapping (**Figure 7A**). A higher mean absolute SHAP value indicated greater influence on the spatial delineation of maize. B12_M05 exerted the strongest effect on model output, with the highest mean absolute SHAP value of 0.038, followed by Pheno_NDVI_Amp (0.033). NDVI_M05 ranks third (0.029), while Pheno_NDVI_Max and LSWI_M06 rank fourth and fifth, respectively, with mean absolute SHAP values of 0.026 and 0.022. All other features, including GCVI_M10, exhibited mean absolute SHAP values below 0.02, indicating minor contributions to model predictions.

The SHAP dependence plots showed that spectral band features sensitive to soil moisture conditions, particularly B12, displayed broad SHAP value ranges with clearly separated positive and negative contribution intervals, reflecting pronounced nonlinear relationships with the model output (**Figure 7B**). In contrast, phenological NDVI-derived features (Pheno_NDVI_Amp and Pheno_NDVI_Max) exhibited SHAP value distributions predominantly concentrated in the positive contribution region, suggesting consistently enhancing effects on maize classification.

**Figure 8:**
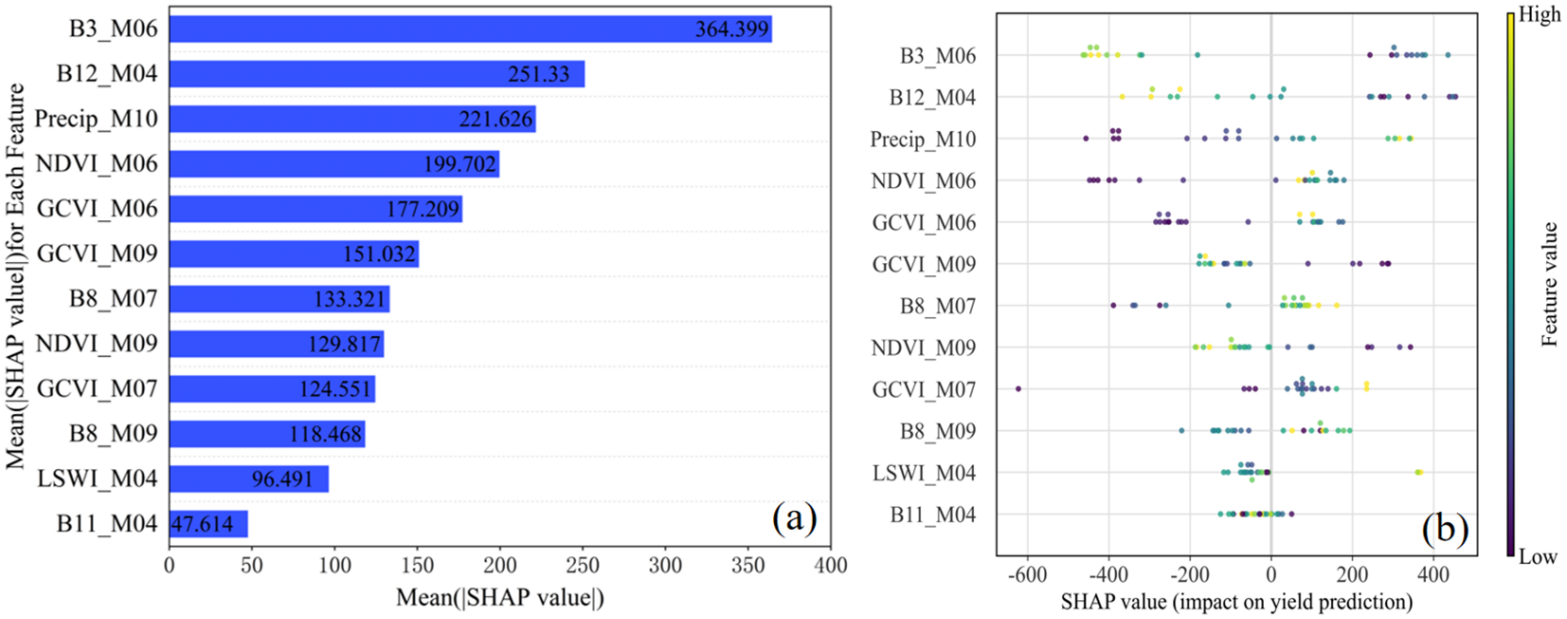
SHAP-based interpretation of feature importance for Histogram-Based Gradient Boosting Decision Tree model. **(A)** Global feature importance ranking, determined by the mean absolute SHAP values; **(B)** SHAP summary plot (beeswarm plot), illustrating the distribution of SHAP values for each feature and their influence on yield predictions, with points color-coded according to feature values (purple: low; yellow: high).

We ranked the input features according to their mean absolute SHAP values for yield estimation (**Figure 8A**). Among all features, B3_M06 exhibited the highest mean absolute SHAP value of 364.399, substantially exceeding all other variables. B12_M04 ranked second at 251.33, followed by Precip_M10 in third place at 221.626. NDVI_M06 and GCVI_M06 occupied the fourth and fifth positions, with mean absolute SHAP values of 199.702 and 177.209, respectively.

The SHAP dependence plots revealed distinct patterns of feature influence (**Figure 8B**). Specifically, band-related features sensitive to soil moisture conditions, particularly B3 and B12, exhibit a broad distribution of SHAP values, spanning both positive and negative intervals. In contrast, the NDVI features demonstrate divergent contributions across phenological stages: NDVI_M06 exerts a consistently positive effect on yield predictions, whereas NDVI_M09 exhibits a predominantly negative contribution.

## 5 Discussion

### 5.1 Adjusting the classification confidence threshold as a key strategy for accurate area estimation

Regional crop mapping often uses default confidence thresholds, assuming strong spectral separability and balanced land cover (Chabalala et al., 2023; Feng et al., 2025; Rusňák et al., 2023). However, in the Loess Plateau’s hilly regions (e.g., Pengyang County), highly fragmented fields and pronounced spectral mixing cause Sentinel-2 pixels to integrate signals from heterogeneous intra-field features such as field ridges, bare soil, rural roads, and natural vegetation edges (Yang et al., 2024). Consequently, a substantial proportion of pixels fall into an ambiguous decision boundary between maize and non-maize classes. In this study, when the RF model employed a lower, adaptive confidence threshold of 0.65, it classified over half of the agricultural plots in the study area as maize (**Figure 3A**), resulting in a severely overestimated maize planting area that deviated markedly from ground-truth statistics. This finding was consistent with Song et al. (2023) and Yanan et al. (2022), overly permissive thresholds amplify spectral mixing noise and bias area estimates (Song et al., 2023; Yanan et al., 2022).

After raising the classification confidence threshold to 0.8, the RF model identified a total of 2.754 × 10^4^ Pengyang County in 2025. This estimate deviates by only 8.8% from the official figure of 3.02 × 10^4^ ha mu reported in the 2025 Statistical Yearbook, a discrepancy well within the accepted error margin for county-scale crop mapping using remote sensing (Zhang et al., 2025). These findings align with those of (Izquierdo-Sanz et al., 2026), who demonstrated that, in fragmented agricultural landscapes, moderately increasing the confidence threshold, while accepting a modest reduction in spatial completeness, effectively suppresses ambiguous classifications arising from edge pixels and small, isolated plots, thereby enhancing the accuracy of areal statistics.

For the purpose of objectively evaluating the spatial pattern quality underlying these area estimates, we computed class-level landscape metrics across all tested thresholds (**Table 5**; **Supplementary Table 2**). At the 0.65 threshold, the Aggregation Index (AI = 82.53%) and Percentage of Like Adjacencies (PLADJ = 70.20%) appear to indicate high spatial contiguity. However, at this overly permissive threshold, non-agricultural pixels, including bare soil, field ridges, rural roads, and grassland edges, are erroneously classified as maize. These misclassified pixels act as bridging pixels that fuse discrete true agricultural patches into artificially large super-patches, thereby inflating AI and PLADJ while severely overestimating the total area (Cai et al., 2022; Meier and Mauser, 2023). As the threshold increases, the monotonic decline in AI, PLADJ, and mean patch area (AREA_MN) does not signal degraded spatial quality (Li et al., 2023); rather, it reflects the progressive removal of these bridging errors and the exposure of true patch boundaries. In this region, where a single 10-m Sentinel-2 pixel typically encompasses 2–5 heterogeneous smallholder plots interspersed with bare soil and ridges, the lower aggregation metrics at higher thresholds represent a more honest representation of the inherently fragmented agricultural mosaic at the pixel scale. Therefore, for county-level crop area estimation in smallholder systems, areal fidelity to official statistics should take precedence over per-pixel landscape aggregation metrics as the criterion for threshold optimization (Fang et al., 2025). These results reinforce the view that, for county-level crop area estimation, confidence threshold tuning should prioritize fidelity to the true regional planting extent rather than strict optimization of per-pixel classification accuracy (Gu and Congalton, 2020).

### 5.2 Agronomic drivers of key spectral and phenological features

In this study, SHAP analysis revealed that May shortwave infrared (B12_M05) ranked first in feature importance for crop mapping, whereas June green band (B3_M06) ranked first for yield estimation. Both features exhibited broad positive and negative contribution intervals in their SHAP value distributions (**Figures 7B**, **8B**), indicating pronounced nonlinear effects. For crop mapping, B12_M05 captured soil moisture conditions during the emergence stage, when canopy coverage is minimal and bare soil signals dominate. For yield estimation, B3_M06 reflected early canopy chlorophyll activity during the jointing stage, while April shortwave infrared (B12_M04) captured pre-sowing soil moisture status (Hosseinpour et al., 2025; Shi et al., 2025; Xia et al., 2026). In dryland farming systems, soil moisture coupled with early vegetative vigor is the principal limiting factor governing seedling emergence rate, early root development, and subsequent biomass accumulation. Elevated B12 reflectance signified soil drought and water deficit, which impose irreversible negative impacts on final grain yield; conversely, elevated B3 reflectance in June indicated sparse vegetation cover and low chlorophyll activity due to poor early growth or moisture stress, thereby undermining yield potential. Reduced B3 reflectance, in contrast, denoted dense early canopy and vigorous chlorophyll activity during the critical jointing period, promoting photosynthetic efficiency and laying a critical foundation for biomass accumulation. These findings aligned with the established agronomic understanding that spring droughts are recurrent across the Loess Plateau and that soil moisture status coupled with early vegetative health during the sowing-to-jointing period was a key determinant of annual yield (Lan et al., 2025; Yu et al., 2026).

Furthermore, the NDVI temporal amplitude (Pheno_NDVI_Amp) played a significant role in crop mapping, with a mean absolute SHAP value of 0.033. This importance stemmed from the distinct phenological trajectory of maize, an annual, tall-stemmed crop with high biomass accumulation. During its growing season, maize exhibited a pronounced NDVI dynamic, starting from bare-soil sowing (NDVI ≈ 0.1), progressing through rapid canopy closure at heading (NDVI ≈ 0.9), and culminating in senescence at maturity (NDVI ≈ 0.3). This caused a temporal NDVI range exceeding 0.8. In contrast, perennial natural vegetation, including forests, grasslands, and shrublands, typically displays much smaller intra-annual NDVI fluctuations, generally remaining below 0.4. Consequently, NDVI temporal amplitude serves as a robust discriminant feature for distinguishing maize from natural vegetation (Han D. et al., 2025; Li Q.et al., 2025; Miao et al., 2026). In yield estimation, NDVI_M06 (NDVI in June) demonstrated a significant positive contribution. June corresponded to the vigorous vegetative growth stage of maize, during which leaf area index expanded rapidly. As a key indicator of canopy greenness, the positive influence of NDVI on yield reflected the central role of photosynthetic activity in driving biomass accumulation (Li X. et al., 2025; Suaza-Medina et al., 2024). Notably, NDVI in September exhibited a negative contribution, a pattern consistent with the physiology of rainfed maize. Excessively high canopy greenness during the grain-filling period may signaled delayed senescence due to late maturity or late-season water stress, under such conditions, prolonged vegetative activity impeded the efficient translocation of assimilates to developing grains (Lan et al., 2025).

### 5.3 Remote sensing–based crop yield estimation

Although SHAP analysis elucidated the natural driving mechanisms underlying crop yield formation, the accuracy of model-based yield estimation for small agricultural plots remained limited by data constraints (Jin et al., 2017). In this study, the HGBDT model effectively captured macro-scale spatial patterns of county-level yield (R² = 0.6, rRMSE = 11.49%); however, low-yield plots tended to be overestimated while high-yield plots are underestimated (**Figures 5C** and **6C**). This bias was especially pronounced in fragmented landscapes (Perich et al., 2023). In the study region, smallholder maize plots typically span less than 0.1 ha, whereas a single Sentinel-2 10-m pixel (0.01 ha) often encompasses 2–5 distinct plots. Consequently, spectral signals from field ridges, bare soil, and adjacent roads were inevitably mixed within each pixel (Izquierdo-Sanz et al., 2026). Moreover, several cultivated areas in this study are situated at high elevations, where microclimatic differences, driven by slope aspect and gradient, are substantial (Yu et al., 2018). However, the ERA5-Land reanalysis data lacked the spatial resolution to capture critical fine-scale heterogeneities, including precipitation interception, radiation gradients, and soil moisture variability between hilltops and valleys (Dai et al., 2023; Han L. et al., 2025). Such cross-scale information loss represents a key source of uncertainty limiting the accuracy of model-based yield estimation in this study.

Furthermore, the lack of smallholder field management information, such as cultivar selection, fertilization practices, and irrigation regimes, further limited the capacity of model to explain yield variability at the small-plot scale (Meng et al., 2021). Despite the practical constraints posed by the scarce farm management data and the inherent mixed-pixel effect in fragmented agricultural landscapes, this study successfully estimated crop yields at the county scale by integrating multi-source features and employing synergistic machine learning modeling. Moreover, SHAP analysis was also employed to elucidate the underlying natural drivers of yield formation. Notably, these limitations reflect a widespread bottleneck in remote sensing, based monitoring of smallholder agriculture at the county scale under current data availability conditions (Masiza et al., 2026).

### 5.4 Limitations and future prospects

This study specifically investigates tree-based ensemble machine learning algorithms, including RF, ET, GBDT, and HGBDT, selected for their empirically demonstrated robustness in modeling fragmented landscapes, where ground reference samples are scarce and remote sensing features are inherently heterogeneous and multi-source (Abate et al., 2025; Weitkamp and Karimi, 2023). Although deep learning models have achieved strong performance in large-scale, homogeneous agricultural settings with abundant labeled training data, their generalizability to smallholder farming systems remains uncertain, particularly under conditions of limited field validation data and high spectral and topographic heterogeneity (Wang et al., 2022). With the aim of advancing methodological rigor and scalability, future work should systematically benchmark the proposed framework against state-of-the-art deep learning architectures under comparable experimental conditions.

## 6 Conclusions

We developed a multi-source feature fusion framework with machine learning to map maize distribution and estimate yield for smallholder farms in the Loess Plateau. Based on systematic multi-model comparison and SHAP-based interpretation, The Random Forest model achieved the optimal overall performance (Accuracy = 0.825, Precision = 0.849, Recall = 0.933, F1-Score = 0.889) for crop mapping. By raising the classification confidence threshold to 0.8, we effectively mitigated spectral mixing noise from fragmented agricultural plots, yielding an estimated maize planting area of approximately 2.754 × 10^4^ ha in Pengyang County. For spatial yield estimation, the HGBDT model achieved acceptable predictive performance at the county scale (R² = 0.6, RMSE = 1.07 t/ha, rRMSE = 11.49%, MAE = 0.86 t/ha), the model reasonably reflected the macro-scale spatial variation in maize yield across Pengyang County. Feature importance patterns diverged markedly between crop mapping and yield estimation. Crop mapping was predominantly driven by spectral and phenological features, particularly Sentinel-2 Band 12 reflectance in May and the amplitude of the NDVI phenological curve. In contrast, spatial yield variation was primarily governed by a different set of features: Sentinel-2 Band 3 reflectance in June, Sentinel-2 Band 12 reflectance in April. Given the limited availability of detailed smallholder field management data, we evaluated the explanatory capacity of purely data-driven models for capturing yield heterogeneity at the small-plot level within a county-scale framework. These findings highlight the potential and limitations of multi-source remote sensing synergy for yield estimation in complex agro-ecological systems. These findings provide a data-driven methodological foundation for refined agricultural monitoring, climate-resilient adaptation strategies, and regional food security assurance.

## Data Availability

The data analyzed in this study is subject to the following licenses/restrictions: Data will be made available on request. Requests to access these datasets should be directed to Hao Li, lihao@iwhr.com.
