# Supplementary material for: A multi-source remote sensing and machine learning framework for maize mapping and yield estimation in fragmented Loess gully regions
Source: Front Plant Sci. 2026 Jul 1;17:1892566. doi: 10.3389/fpls.2026.1892566 (PMC13369130; doi:10.3389/fpls.2026.1892566)
Supplement: Supplementary file 1 [file Table1.docx]

**Supplementary information**


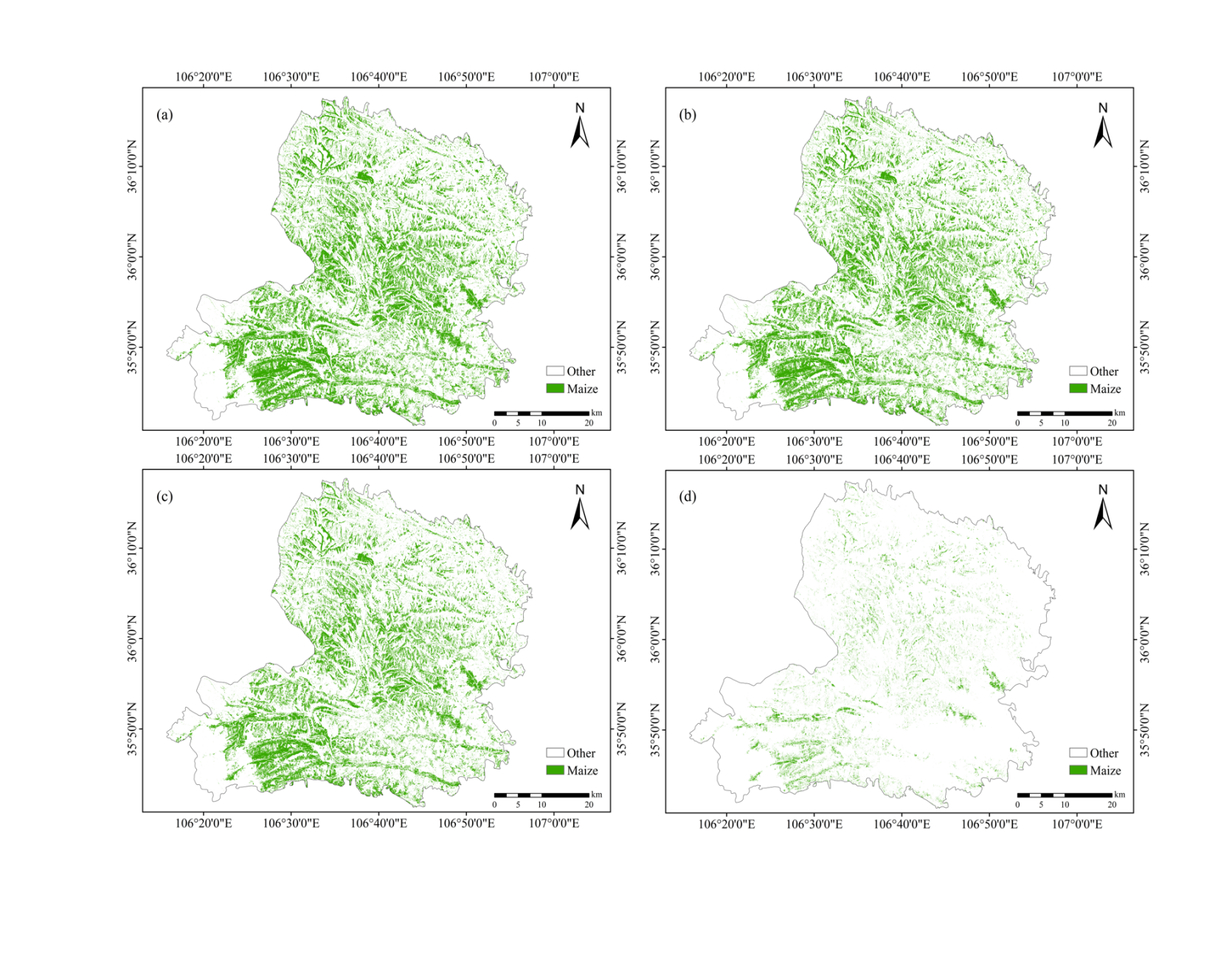


**Supplementary Figure S1.** Spatial distribution extraction results obtained under varying threshold values using Random Forest (RF). (a) 0.50; (b) 0.55; (c) 0.60; (d) 0.85.

**Supplementary Table S1** Sentinel-2 overpass times corresponding to three in-situ ground measurements

| Date | Time | Orbit No. |
| --- | --- | --- |
| July 24 | 11：48：24 | 61 |
| July 24 | 11：48：38 | 61 |
| July 29 | 11：48：02 | 61 |
| July 29 | 11：48：17 | 61 |
| August 25 | 11：48：16 | 61 |
| August 25 | 11：48：31 | 61 |
| August 28 | 11：48：03 | 61 |
| August 28 | 11：48：18 | 61 |
| September 27 | 11：48：02 | 61 |
| September 27 | 11：48：16 | 61 |

**Supplementary Table S2** Landscape metrics of maize classification under varying confidence thresholds

| Threshold | CA (ha) | Relative Error (%) | AREA_MN (ha) | PLADJ (%) | AI (%) |
| --- | --- | --- | --- | --- | --- |
| 0.50 | 8.122 × 10^4^ | +168.9 | 0.87 | 77.33 | 87.24 |
| 0.55 | 7.337 × 10^4^ | +142.9 | 0.72 | 75.36 | 85.98 |
| 0.60 | 6.634 × 10^4^ | +119.7 | 0.63 | 73.55 | 84.79 |
| 0.85 | 1.188 × 10^4^ | -60.7 | 0.12 | 50.07 | 66.79 |
